# Numerical Investigation of Aerodynamic Noise Reduction of Nonpneumatic Tire Using Nonsmooth Riblet Surface

**DOI:** 10.1155/2020/4345723

**Published:** 2020-03-14

**Authors:** Haichao Zhou, Zhen Jiang, Jian Yang, Huihui Zhai, Guolin Wang

**Affiliations:** ^1^School of Automotive and Traffic Engineering, Jiangsu University, Zhenjiang 212013, China; ^2^School of Automotive Engineering, Zhenjiang College, Zhenjiang 212000, China

## Abstract

Unlike conventional pneumatic tires, the nonpneumatic tires (NPT) are explosion proof and simple to maintain and provide low rolling resistance. At high vehicle speeds, however, the complex airflow produced by the open flexible-spoke structure of NPT yields high aerodynamic noise, which contributes to sound pollution in the vehicular traffic environment. Inspired by the idea that a nonsmooth riblet structure can affect fluid flow and offer noise reduction, the analyses of the effect of the nonsmooth riblet surface on the aerodynamic noise of an NPT and noise reduction mechanism were presented in this paper. First, computational fluid dynamics (CFD) was used to analyze the surface pressure coefficient characteristics of a smooth flexible-spoke tire rolling at a speed of 80 km/h and subsequently validating the numerical simulation results by comparing them with published test results. Secondly, large eddy simulation (LES) and the Ffowcs Williams–Hawkings (FW-H) method were, respectively, used to determine the transient flow and far-field aerodynamic noise. Then, the mechanism of noise reduction was investigated using a vortex theory. Based on the vortex theory, the positions and strengths of noise sources were determined using the Lamb vector. Finally, according to the fluid boundary layer theory, a nonsmooth riblet surface was arranged on the surface of the spokes, and the influences of the riblet structure parameters, including size, position, and direction, on aerodynamic noise were analyzed. Based on the vortex theory, it was found that the nonsmooth riblet structure can reduce the Lamb vector, suppress the generation of flow vortices, decrease acoustic source strength, and effectively decrease noise up to 5.18 dB using the optimized riblet structure. The study results provide a theoretical basis for the structural design of a new low-noise NPT.

## 1. Introduction

Since pneumatic tire invention in 1888 by Dunlop, it has been primacy choice for use in automobiles, but this comes with several drawbacks, including susceptibility to catastrophic damage, complex manufacturing process, and air leakage during driving and its required air pressure limits the free design space of a car [1]. Unlike a conventional tire, in the nonpneumatic tire (NPT), the need for a tire to be inflated with air to support vehicle weight is eliminated since a spoke or hub is used. This has several advantages, including low rolling energy loss with the use of low-viscoelastic-energy-loss materials, low mass, and low contact pressure [2]. NPT adopts a spoke and tread structure composed of a polyurethane material. The geometric structure and material performance parameters have great influences on rolling resistance, vertical stiffness, and contact pressure of the wheel [3]. Under the same load, the contact footprint of an NPT is rectangular, whereas it is oval for a pneumatic tire. Moreover, NPT do not depend on air to support the weight of a vehicle, and the flexible spokes result in high tire elasticity. In addition, NPT boast antibarding and explosion resistance characteristics and offer excellent driving safety.

The spokes of NPT are generally arranged circumferentially. Due to the fact they are discontinuous, it has open spaces between them. The width of the spokes is equal to the lateral width of the tire. The main reason for this arrangement is that the lateral deformation of NPT is small when subjected to lateral load [4]. When the rolling speed is high, the spokes cut the air speedily, resulting in a complex airflow and generating a loud aerodynamic noise. Heo et al. studied the aerodynamics of an NPT under static and rolling conditions using the steady Navier-Stokes method [5]. Their results showed that an open-spoke structure always results in a more complicated flow-field distribution compared with that of the conventional pneumatic tire. This may be one reason for the limited application of the NPT in a vehicle tire design. NPT aerodynamic noise is ubiquitous in vehicle flow field, especially in electric vehicles. The aerodynamic noise of NPT has become the key challenge in clean energy car research. However, relatively few reports have discussed aerodynamic noise reduction methods for the NPT design.

Because aerodynamic noise originates from fluid motion, controlling the method of flow to alter the aerodynamic noise generation is now a generally accepted strategy. The bionics provides new ideas and technologies for noise reduction in engineering applications. Boundary laid control utilizing riblets with different cross sections has received considerable attention through the use of riblets. One biological feature that is yet to be used in engineering innovations is the silent flight of barn owls. The adaption of barn owl wings to silent flight is due to the wind geometry, where the wing feather has the serration structures in the leading edge [6, 7]. And this serration structure of owl-specific characteristics has an influence on the noise emission of the wing. Oerlemans et al. confirmed that a trailing-edge riblet structure can effectively reduce the trailing-edge noise of a wind turbine blade after obtaining a total sound pressure reduction of 3 dB [8]. Shi et al. analyzed the effect of bionic serrated structures on the aerodynamic noise of a circular cylinder and point out that the aerodynamic noise can be reduced in the case of the cylinder with serrated structures [9]. In addition, bionic nonsmooth structures are applied for noise reduction in high-speed trains [10], automobile bodies [11], aircraft designs [12], and tire patterns [13], and studies have indicated good prospects for flow engineering applications.

Inspired by the awareness that the riblet structure can reduce aerodynamic noise, we propose a bionic method, wherein the riblet structure is arranged on the spoke surface of NPT as one of the flow control methods to reduce the aerodynamic noise. First, a three-dimensional geometric model of the NPT is established, and its surface pressure coefficients at 80 km/h are presented using computational fluid dynamics (CFD). A comparison with published test results is used to validate the NPT model. Second, the unsteady flow on the basis of the large eddy simulation (LES) method and aerodynamic noise equation of the Ffowcs Williams–Hawkings (FW-H) equation is chosen to predict the sound pressure level, while the distribution, position, and strength of the acoustic sources of the NPT are identified using the vortex theory. Third, a nonsmooth riblet structure is established on the flexible spokes based on a fluid boundary layer theory, and the effect of the riblet structure parameters; size, position, and direction, on the far-field sound pressure is analyzed. Finally, a comparison of the fluid-field parameters using the vortex theory is used to investigate the noise reduction mechanism.

## 2. Numerical Details

### 2.1. Model Geometry

**Figure 1:**
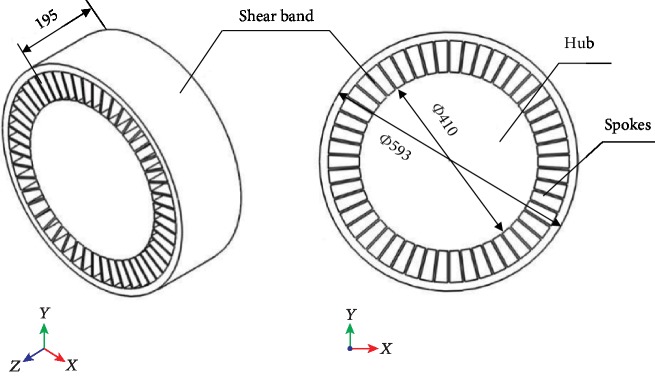
Geometry and sizes of NPT (mm).

The detailed geometric parameters for this NPT model, with reference to [14], are shown in Table 1. The model structure mainly includes a rigid hub on the inner side, a smooth surface shear band on the outer ring, and a flexible spoke connecting the hub and the shear band (Figure 1). Because this paper focuses on exploring the influence of the nonsmooth riblet spoke structure on the flow field and aerodynamic noise of an NPT, the deformation characteristics of the tire-road contact is addressed. Consequently, flexible spokes with a fine curvature and original bearing function are simplified as straight spokes in the radial direction. The rigid hub on the inside is simplified to a rigid plane.

### 2.2. CFD Model

**Figure 2:**
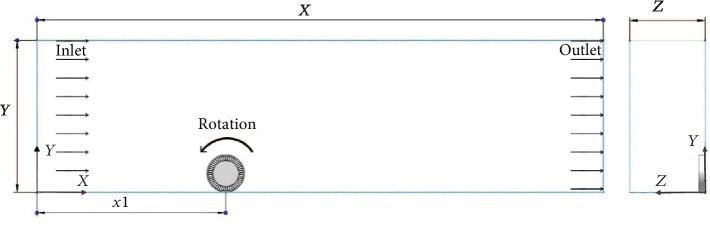
Computational domain size of the flow field around an NPT.

Because the NPT model is symmetric along the center plane at *z* = 0, only half of the model is used for numerical calculation. The CFD model and domain sizes are shown in Figure 2. The length of the model (X-direction) is 9.0 m, the height (Y-direction) is 2.4 m, the width (Z-direction) is 1.2 m, and the distance (x1) from the inlet surface to the center of the contact area is 2.0 m.

**Figure 3:**
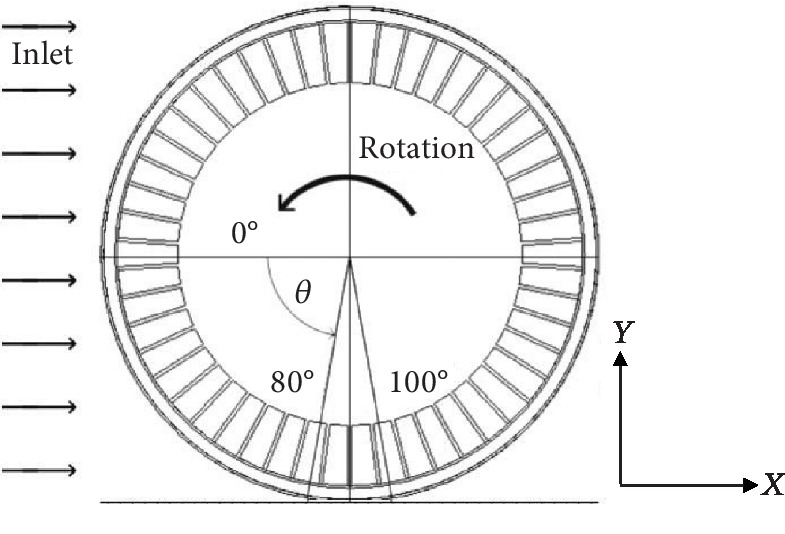
Definition of contact angle.

**Figure 4:**
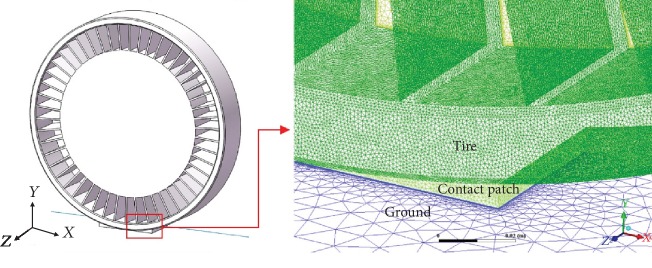
Flow-field meshing around the contact area.

Axon et al. [15] found that, close to a tire, the geometric shape of the tire-road contact significantly affects the aerodynamic characteristics. The contact angle, defined as 0°, is at the front end of the tire and gradually increases along the rolling direction (Figure 3). They also indicated that the length of the tire-road contact corresponds to a contact angle of 80° to 100°. When a vertical load of 3000 N is applied on the NPT, there will be a platform deformation in contact area. In order to reflect the platform deformation, a block is used at the contact angle to indicate the contact shape (Figure 4). According to the contact length of the NPT obtained from Abaqus simulation, the length of the flat block is 16 mm, and the corresponding contact angles at the front and rear ends are 80° and 100°, respectively. Because of the existence of the block, a low-quality grid maybe generated in the contact angles, and the depth of the flat block is set as 1 mm.

### 2.3. Boundary Conditions

The boundary conditions are set as follows: inlet velocity of 22.22 m/s (80 km/h) along the positive X-direction, which is applied to match the reference Reynolds number of 9.17 × 10^5^ considering the NPT geometry. A zero-pressure condition is prescribed at the outlet, and a symmetry condition is around the z = 0 plane. The ground is treated as a nonslip wall with constant moving speed along the positive X-direction, while the NPT surface is taken as a nonslip wall with angular velocity (74.94 rad/s) corresponding to the translation speed of the road, and the remaining surfaces are set to zero-pressure condition.

The calculated model comprises a bound layer and tetrahedral elements derived from the Hypermesh software. To accurately simulate the flow of the near wall, a boundary layer is provided on the tire surface of each mesh.  Table 2 shows the grid parameter settings under different schemes and the corresponding computational time. The steady computer results of pressure coefficients on the shear band at the *z* = 0 plane are used as indicators to conduct the sensitivity analysis of the different grid generation schemes.

A steady simulation calculation is performed using ANSYS-Fluent software in a double-precision mode. One of the most common two-equation Reynolds average Navier-Stokes (RANS) models is the realizable *k*-*ε* model, and the transport equations for the realizable *k*-*ε* model are given in detail in [16, 17]. The solution algorithm for the simulation was based on the well-known SIMPLE algorithm for the iterative solution of the steady RANS equations, and the second upwind scheme was employed for the discretization for all variables.

The pressure coefficient is defined as

(1) $$\begin{array}{c} C_{p}=\frac{p_{i}-p_{r}}{p_{0}-p_{r}}=\frac{p_{i}-p_{r}}{\left(1/2\right)\rho U_{r}^{2}}. \end{array}$$

**Figure 5:**
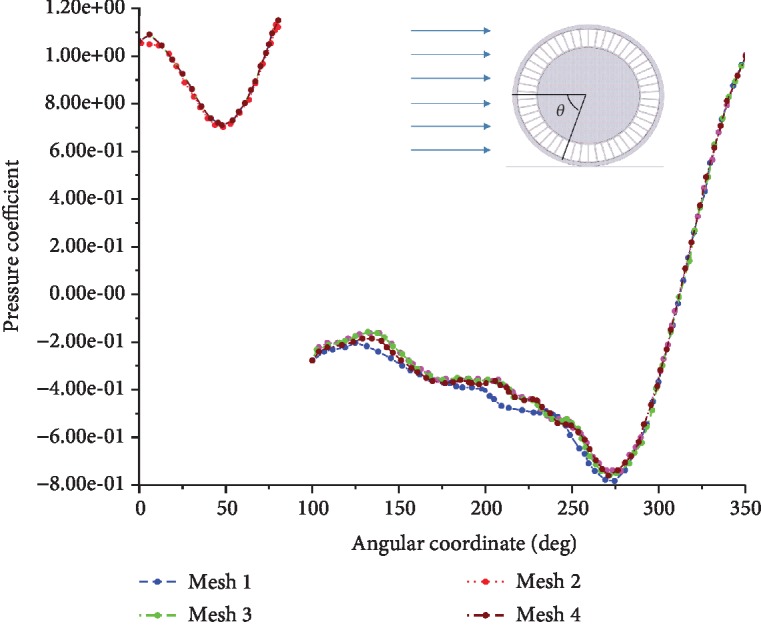
Distribution of pressure coefficient at the *z* = 0 plane under different meshes.

Figure 5 shows the pressure coefficient (*C*_*p*_) characteristics of the symmetry plane (*z* = 0 plane) under each grid scheme. Note that, because of the existence of the contact patch, the corresponding *C*_*p*_, where *θ* is between 80° and 100°, does not exist in the figure. The *C*_*p*_ of Grid 3 coincides well with that of Grid 4. The *C*_*p*_ differs slightly between Grid 2 and Grid 3 in *θ* = 100°–180° and *θ* = 210°–270°. For the *C*_*p*_ of Grid 1, there is a significant deviation of the two curves of Grid 1 and Grid 3 it *θ* = 100°–270°. Therefore, Grid 3 or Grid 4 can accurately reflect the *C*_*p*_ of NPT for the flow-field simulation. Because of its accuracy and computational efficiency, the Grid 3 scheme is used as the grid quality benchmark for subsequent calculation.

**Figure 6:**
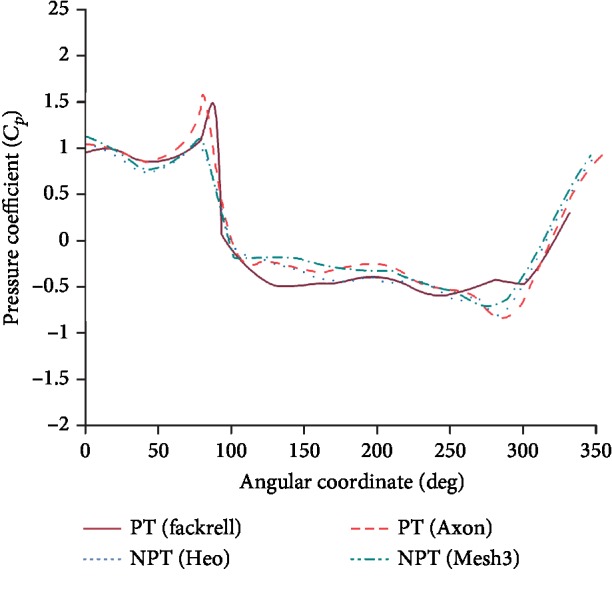
Comparison of pressure coefficients from other literatures.

To validate the accuracy of the CFD model, comparisons of pressure coefficients from the published literature with the simulation results of this study are presented in Figure 6. The general tendency of the simulation result used by Grid 3 is consistent with the test results for a pneumatic tire obtained by Fackrell [18], simulation results for a pneumatic tire obtained by Axon et al. [15], and simulation results for an NPT by Heo et al. [5]; however, the peak pressure coefficients are different. One explanation for the discrepancy is the difference in the geometric outline of the tire.

## 3. NPT Aerodynamic Noise Simulation

### 3.1. Method for Aerodynamic Noise Calculation

Based on the steady computer results in Chart 2, the converged result of the steady calculation is used as the original flow field, and the LES method is used for unsteady calculation. First, the time step is set to 0.001 s for iterative computations of 2000 steps. The total computing time is 2 s, meaning that the total time is 5 times that of the domain airflow. After the 2000-step iterations, the flow field tends to be relatively stable. Next, the time step is reduced to 0.000025 s, and the unsteady field is recalculated with 2001 steps. At the same time, the acoustics model is open. According to the Nyquist sampling theorem Δ*t* = 1/2*f*_max_, where Δt is the time step and *f*_max_ is the maximum acoustic frequency. The highest sound frequency that can be obtained from these samples is 20 kHz. The recording of the flow-field pressure data is selected from the 2001 samples, corresponding to a duration of 0.05 s, that is, the lowest frequency of 20 Hz.

The FW-H equation based on Lighthill's acoustic analogy theory is used to predict the far-field sound pressure level (SPL). The FW-H equation can be expressed as

(2) $$\begin{array}{c} \frac{\partial^{2}p^{\prime }}{c_{0}^{2}\partial t^{2}}-\nabla^{2}p^{\prime }=\frac{\partial }{\partial t}\left\{\left[\rho_{0}v_{n}+\rho \left(u_{n}-v_{n}\right)\right]\delta \left(f\right)\right\}-\frac{\partial }{\partial x_{i}}\left\{\left[\tau_{ij}n_{j}+\rho u_{i}\left(u_{n}-v_{n}\right)\right]\delta \left(f\right)\right\}+\frac{\partial^{2}}{\partial x_{i}\partial_{j}}\left[T_{ij}H\left(f\right)\right], \end{array}$$

where *p*′ is the far-field sound pressure, *p*′ = *p* − *p*_0_; *p*, *ρ* and *p*_0_, *ρ*_0_ are the pressure and density of the fluid before and after perturbation, respectively; c_0_ is the speed of sound; u_*n*_ is the fluid velocity component; v_*n*_ is the surface velocity component; *T*_ij_ is the Lighthill's stress tensor; *δ*(f) is the Dirac function; and *H*(f) is the Heaviside function, where f represents the body surface as the function of *f*(*x*, *t*) = 0, in which f≺0 and f≻0 refer to the inside and outside of the rigid body, respectively. In this current research, the tire surfaces, except the hub, are defined as acoustic sources.

The SPL is used to evaluate the aerodynamic noise level in decibels, calculated as

(3) $$\begin{array}{c} \text{SPL}=20\mathrm{lg}\frac{p_{e}}{p_{\text{ref}}}, \end{array}$$

where *p*_*e*_ is the effective pressure value and *p*_ref_ is the reference pressure value, which is usually set as 2 × 10^−5^ Pa.

The total SPLs are used to evaluate the synthetic sound pressure level in all the measuring points, calculated as

(4) $$\begin{array}{c} \text{SPLs}=10\mathrm{lg}\left(10^{\frac{L1}{10}}+10^{\frac{L2}{10}}+\cdots +10^{\frac{Li}{10}}\right), \end{array}$$

where *L*_*i*_ is the SPL in different measuring points, where *i* = 1, 2, ⋯, 5.

**Figure 7:**
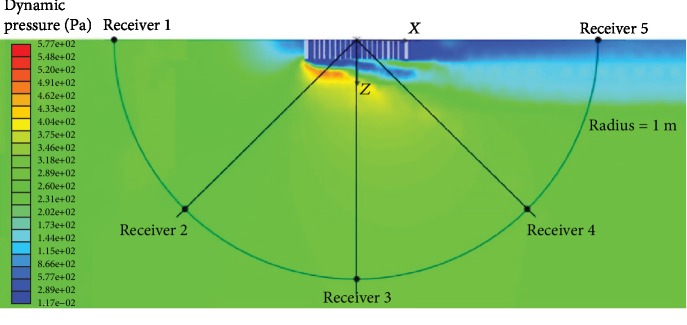
Dynamic pressure distribution at the *y* = 0.1 m and the position of the five sound-measuring points.

To eliminate the interference of near-field turbulence with the sound signal, the position of the measuring point is selected to be within a relatively stable flow field.  Figure 7 shows the dynamic pressure distribution on a horizontal reference plane at a height of 0.1 m from the ground. The five measuring points are equally spaced on a semicircle with a radius of 1 m. Their positional coordinates are listed in Table 3 (the coordinates of the NPT center are *x* = 2.0 m, *y* = 0.2965 m, and *z* = 0 m).  Table 4 lists the different SPLs of the original NPT at each measuring point. The highest SPL is near the rear end with respect to the rolling direction, whereas the minimal SPL is at the lateral position. The results show that the acoustic field has obvious directivity.

### 3.2. Howe's Vortex Sound Theory

Howe [19] defined the stagnation enthalpy Equation (5) as the acoustic variable in isentropic low-Mach-number flow:

(5) $$\begin{array}{c} B=\int \frac{dp}{\rho }+\frac{1}{2}\left(\frac{\partial \emptyset }{\partial x_{i}}\right). \end{array}$$

Taking incompressibility of the fluid into consideration than those in Powell's equation, a final vortex sound equation can be obtained:

(6) $$\begin{array}{c} \left\{\frac{D}{Dt}\left(\frac{1}{c_{0}^{2}}\frac{D}{Dt}\right)+\frac{1}{c_{0}^{2}}\frac{D_{v}}{D_{t}}\frac{\partial }{\partial x}-\nabla^{2}\right\}B=\nabla ∙\left\{\omega \times v-T\nabla S\right\}-\frac{1}{C_{0}^{2}}\frac{D_{v}}{D_{t}}∙\left\{\omega \times v-T\nabla S\right\}. \end{array}$$

If we consider the isentropic and low-Mach-number flow, where the only relevant source term is the Powell dipole ∇∙(**ω** × **v**) on the right side of Equation (5), it can be degenerated into the form of a convective wave equation:

(7) $$\begin{array}{c} \left(\frac{1}{C_{0}^{2}}\left(\frac{1\partial }{\partial t}+U∙\frac{\partial }{\partial x}\right)-\nabla^{2}\right)p=\rho \nabla ∙\left(\omega \times v\right). \end{array}$$

Equation (7) can be used to consider the interaction between the mean flow and the wave propagation, and Equation (7) can be considered an appropriate convective vortex sound equation.

The left side of Equation (7) describes the propagation of sound waves in a nonuniform fluid. The right side of Equation (7) represents a vortex source. For isentropic low-speed flow, the divergence of the Coriolis acceleration experienced by the fluid is the basic factor that causes the flow to occur. Its physical meaning is that the sound produced by the tensile deformation of the vortex is in the velocity field. That is, the aerodynamic noise is derived from the stretching and rupturing of the vortex. For a steady and flawless flow, the total enthalpy is constant; this means that there is no sound generation in this fluency flow. It can be seen that the vortex theory relates the airflow radiation noise to the magnitude of vortices. As long as the magnitude, change, and motion of the vortices in the flow field are clarified, the radiated sound field can be analyzed.

Therefore, the right side of Equation (7) can be defined as the Lamb vector, as shown in Equation (8), to indicate the strength and position of the sound source.

(8) $$\begin{array}{c} \text{Lamb}=\nabla \cdot \left(\omega \times v\right). \end{array}$$

To analyze the strength and position of the sound source, the unsteady flow-field results of the NPT are imported into the Tecplot 360 software, and the Lamb vector is obtained using the custom function.

**Figure 8:**
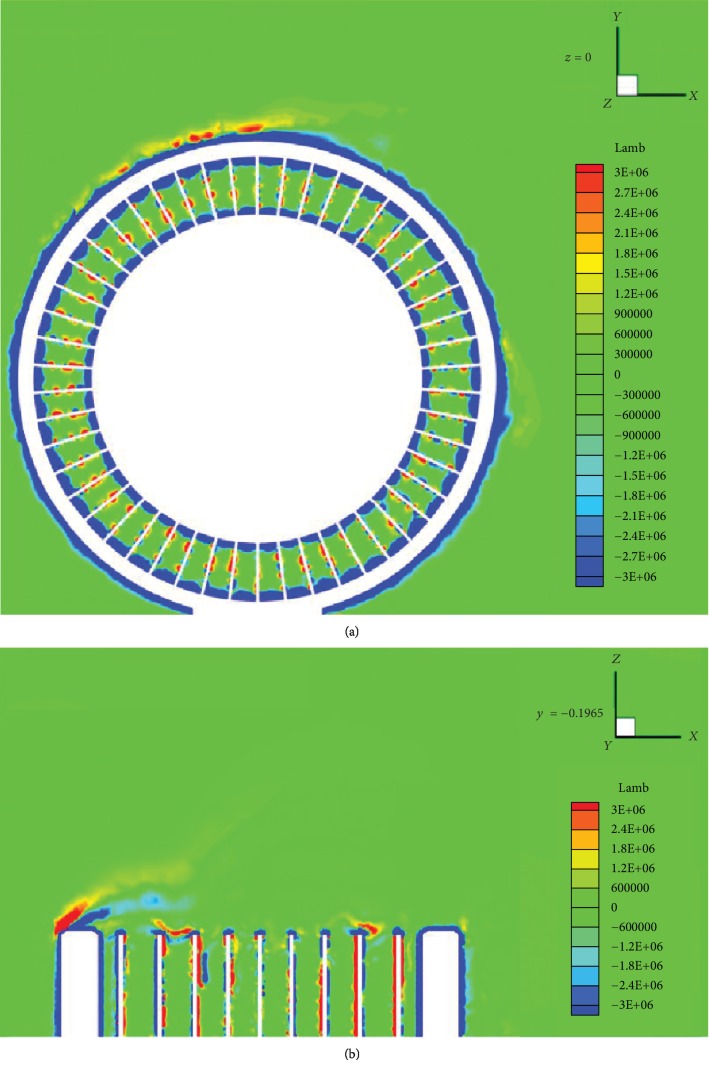
Lamb vector contours of the original NPT on planes *z* = 0 m (a) and *y* = −0.1965 m (b).

The Lamb vector fields of the lateral symmetry plane *z* = 0 and horizontal reference plane *y* = −0.1965 are shown in Figures 8(a) and 8(b), respectively. As shown in Figure 8(a), the Lamb vector's extreme region is concentrated on either side of the shear band and appears at a hub nearby. When the contact angle is in 120°–270°, the Lamb vector magnitude is maximized, and the swirling intensity is the strongest. At the same time, the Lamb vector mainly occurs on either side of the spokes and is more visible in the middle of the spokes.  Figure 8(b) shows that the Lamb vector is concentrated at the extreme region of the spoke openings, and the magnitude of the Lamb vector on the shear-band edge of the windward side is higher than that of the leeward side. Moreover, the magnitude of the Lamb vector at the nearby spokes on the leeward side is higher than that on the windward side. According to vortex sound theory analysis, the flexible spokes are the main aerodynamic noise source of the NPT.

## 4. Effect of Nonsmooth Riblet Structure on NPT Aerodynamic Noise

### 4.1. Design of Nonsmooth Riblet Structure

Because the spokes are the primary source of noise, to reflect the nonsmoothness of living creatures, a series of parallel nonsmooth riblet structures are placed on the surface of the spokes. To prevent damage to the spokes' structure, convex rather than concave riblet designs are preferred. Because noise reduction by the nonsmooth surface affects the flow motion in the boundary layer, the dimensions of the riblet structures are set below the flow boundary layer thickness; which depends on the Reynolds number and characteristic length, as shown in the following equation ^[^20^]^:

(9) δl=0.38lRe1/5,

where *l* is the characteristic length of the tire. In this paper, the outer diameter of the NPT is 0.593 m, and the air flow velocity is 80 km/h; therefore, the Reynolds number (Re) is 9.17 × 10^5^. The estimated boundary layer thickness is approximately 14.5 mm.

### 4.2. Influence of Position and Direction of Riblet Structure on Aerodynamic Noise

**Figure 9:**
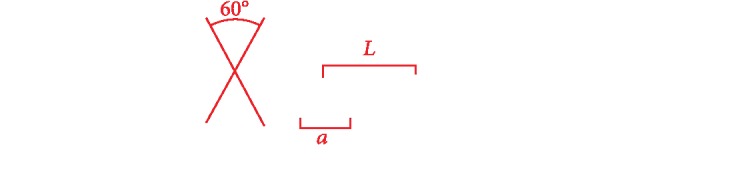
Cross-sectional view of the nonsmooth riblet surface.

**Figure 10:**
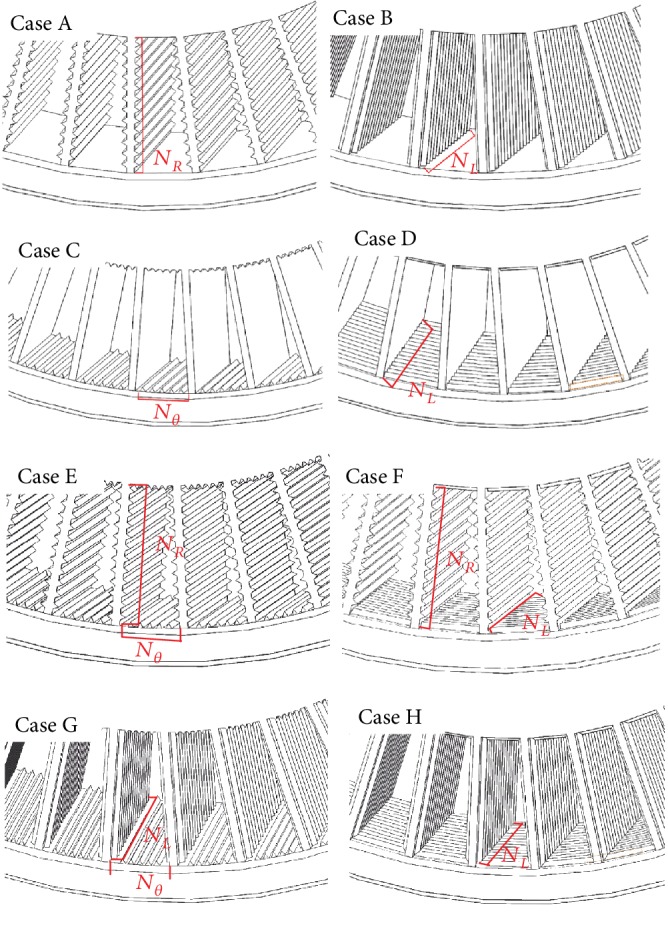
Eight design forms for the riblet structure in the spokes.

Because the spokes are circumferentially discontinuous, numerous cavities are formed in the NPT spokes. Similar to rotating machinery, the rotation of an NPT means the rotation of the airflow within these cavities; simultaneously, the spokes cut air, and the externally oriented flow mainly meets and interacts with the cavity airflow at the edge of the spokes, resulting in complicated airflow. With this complexity, the position and direction of the riblet structure may influence the air motion resulting in different SPLs. To explore a reasonable riblet structure, it is assumed that the cross section of one riblet unit is an equilateral triangle with a side length (*a* = 3 mm) and that the spacing *L* of two adjacent riblet units is twice the side length, as shown in Figure 9. The riblet structures can be placed on the front and rear surfaces of the spoke, that is, the inner surface of shear band and the outer surface of hub; the arrangement may be radial, circumferential, or lateral on either surface. Thus, eight design forms were designed in this paper, as shown in Figure 10; Schemes A–D are basic schemes, and E–H are different combinations of the four basic schemes.  Table 5 details the dimensional parameters of all schemes. The terms *N*_*L*_, *N*_*R*_, and *N*_*θ*_ seen in Table 5, respectively, indicate the lateral, radial, and circumferential numbers of a single-spoke cavity. Because the NPT model is mirrored in the lateral (Z) direction, NL is half the number of riblets in the lateral direction.

The total SPLs, based on the analysis in Section 3.1, of various riblet design schemes are shown in Table 6. The variable Δ*L*_*p*_ indicates the difference between each scheme and the original model; a negative value indicates that the model offers a noise reduction compared with the original model, and a positive value indicates increased noise.

As seen from Table 6, Scheme B, the model in which radially convex riblet structures are arranged on the spokes offers up to 5.1 dB of noise reduction, the largest noise reduction achieved by any scheme. Although laterally arranged riblets on the spokes (Scheme A) achieve a slight noise reduction, riblets placed on the surface of the hub and shear band (Scheme D) do not reduce noise. Schemes C and G even increased the noise. A comparison of three noise-reducing schemes (B, D, and H) reveals that a simple superposition of basic schemes does not always produce greater noise reduction than a basic scheme.

### 4.3. Influence of Riblet Structure Size on Aerodynamic Noise

The analysis of the influence of the size of the riblet structure in Scheme B on the tire's aerodynamic noise is conducted. The SPL of the aerodynamic noise is compared for unit changes in the riblet length of the regular triangular section within the range of 1–5 mm. The maximum length tested is 5 mm because the spoke itself has a thickness of 4.6 mm. The addition of an oversized convex structure can significantly affect a tire's structure and cause deformation under loading. These design schemes are numbered B1–B5 according to the length of the triangular cross section.  Table 7 lists the differences between the total SPLs of the five schemes compared with the original model: Δ*L*_*p*_.

As seen from the Table 7, the size of the riblet structure considerably affects the aerodynamic noise. A riblet structure with a side length of 3–4 mm can effectively reduce aerodynamic noise. This result proves the feasibility of using the riblet nonsmooth surface structure with appropriate parameters to effectively reduce the aerodynamic noise of the NPT. However, side lengths that are too long or short diminish the noise reduction effect and can even increase the noise level.

### 4.4. Noise Reduction Mechanism of the Nonsmooth Riblet Structure

**Figure 11:**
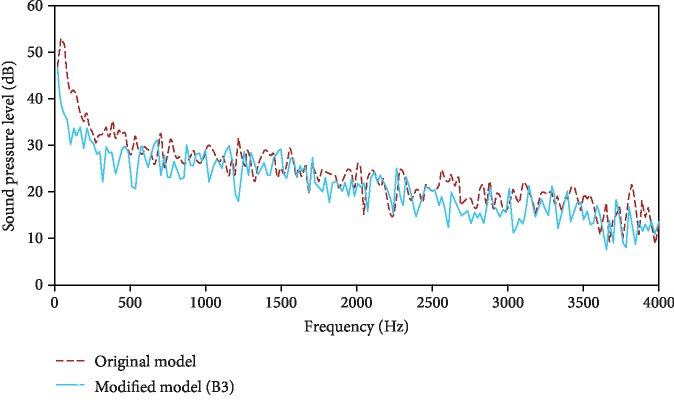
Noise comparison of the original model and modified model (Scheme B3).

Section 4.3 concludes that the noise reduction level of Scheme B3 was the greatest.  Figure 11 shows a noise spectrum comparison of Scheme B3 with the original NPT model. The overall spectrum trends for the two models are the same. The SPL gradually decreases as the frequency increases, but at most frequencies, the SPL of Scheme B3 (nonsmooth riblet structure) is lower than that of the original model. The reduction is greatest in the frequency bands of 20~600 Hz and 2000~3500 Hz.

**Figure 12:**
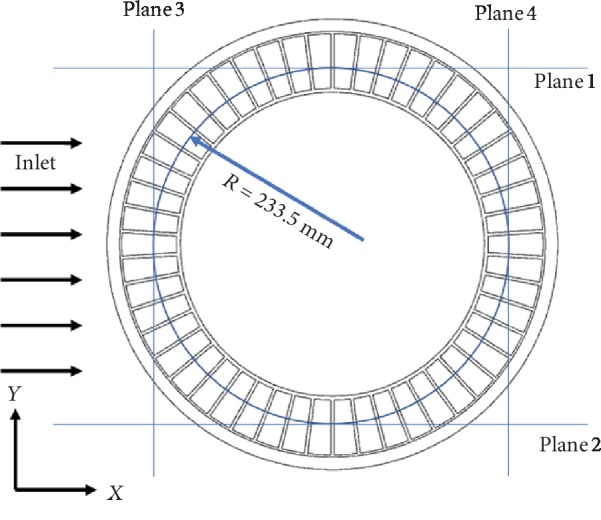
Positions of reference planes 1–4.

**Figure 13:**
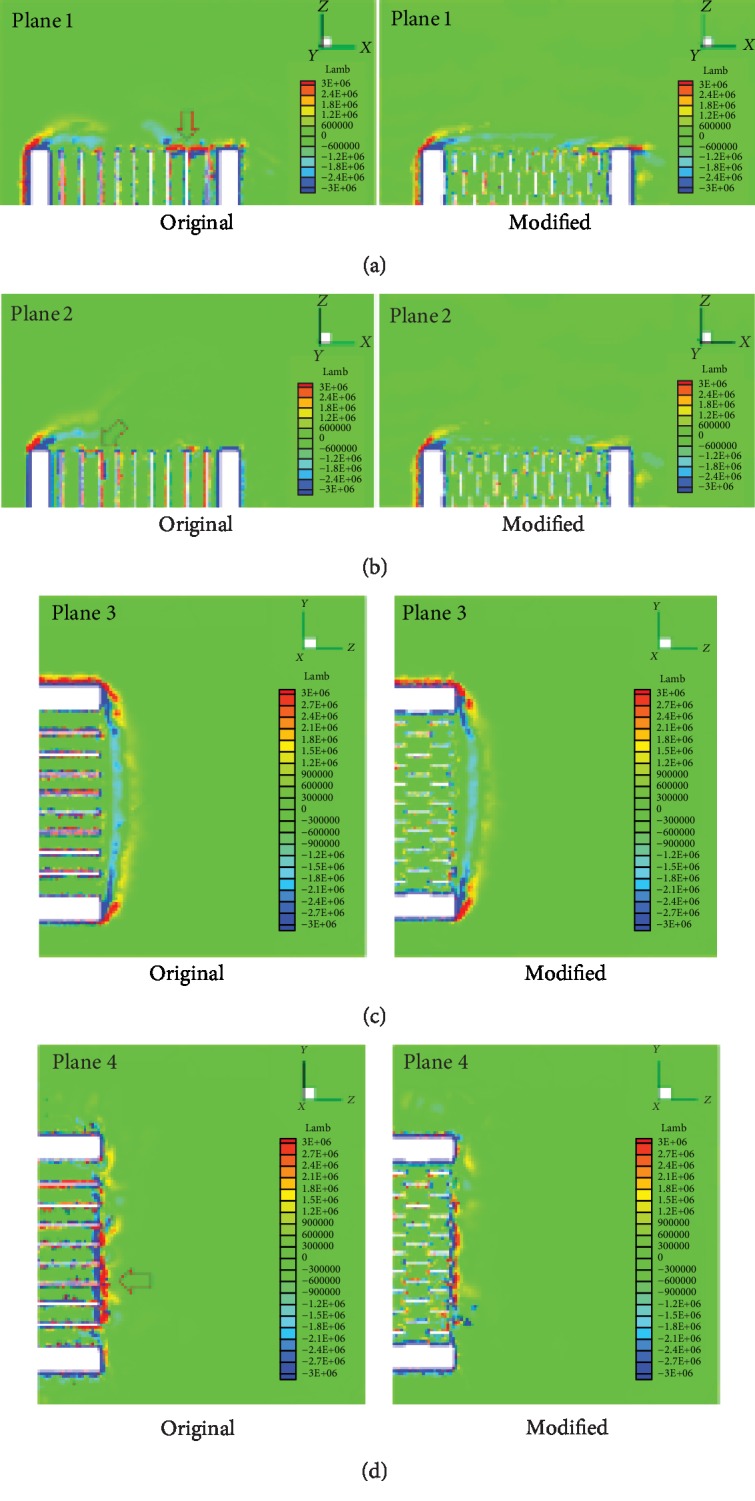
Comparison of the Lamb vectors of the original NPT model and the modified model with Scheme B3 on the four reference planes.

The effects of the flow-field distribution feature of the nonsmooth riblet structure on the aerodynamic noise of the NPT are analyzed as follows: the Lamb vector distributions inside the spoke cavity and nearby shear band are explored, and a mechanism of noise reduction is proposed. Reference planes 1–4 are defined as shown in Figure 12. Reference planes 1 and 2 are horizontal planes with vertical positions of *y* = −0.2965 and *y* = 0.2965; reference planes 3 and 4 are vertical planes with horizontal positions of *x* = −0.2335 and *x* = 0.2335.  Figure 13 shows a comparison of the Lamb vectors on the four reference planes of the original NPT model and the modified model using Scheme B3 at 2.2625 s, the time at which fluid motion reaches a relatively stable state.

Figure 13 also reveals that the extreme values of the Lamb vector are concentrated at the openings of the discontinuous spoke cavities. This means that the flow vortex is mainly generated near the opening, and all openings are aerodynamic noise sources.

Lamb vector extrema with band-like distributions are observed near the openings of reference planes 1, 2, and 4. However, compared with the original NPT model, the Lamb vector of the modified model using a riblet surface is considerably lower, and the band-like distribution is broken. This reduction is most evident in planes 1 and 4. On plane 3, although the Lamb vector on windward and leeward sides of the NPT outer edge does not differ; that near the spokes is markedly reduced. According to the synthetic analysis of the Lamb vector on the four reference planes, the spokes with a nonsmooth riblet surface decrease the Lamb vector, flow strength, and noise strength. The nonsmooth riblet structure plays an important role in weakening the flow vortex. The riblet surface breaks the large vortices of the spoke cavity into smaller vortices, thereby suppressing the fluid vortices produced and ultimately reducing the aerodynamic noise of the NPT.

**Figure 14:**
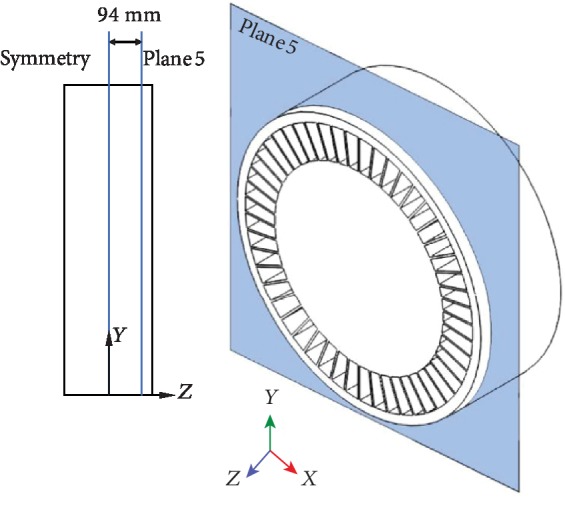
Location of reference plane 5.

To better observe the effect of the nonsmooth riblet structure on air flow and the Lamb vector, another longitudinal reference plane, plane 5, perpendicular to the *z*-axis, 94 mm from the mirror plane of the NPT and 3.5 mm from the lateral edge, as shown in Figure 14, is considered.

**Figure 15:**
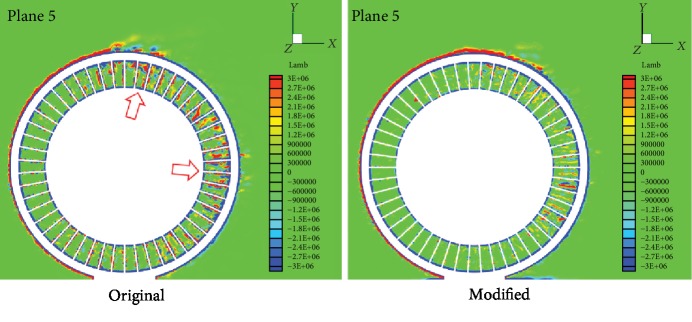
Comparison of the Lamb vectors between the original model and the modified model on plane 5.

Figure 15 compares the Lamb vector distribution of the original model and the modified model in reference plane 5. In plane 5, the Lamb vector at the outer edge of the NPT does not change substantially with modification, but that on the top outer edge decreases lightly.

For the air in the cavity at the rear end of the rolling direction, the Lamb vector near the spokes with the riblet surface is significantly reduced, especially more pronounced in the area marked by the red arrow. The analysis of Figures 13 and 15 indicates that the nonsmooth riblet structure arranged on the spoke surface can reduce aerodynamic noise by suppressing the fluid vortex.

## 5. Conclusion

1. A CFD-analyzed NPT model is established, and its validation is achieved by a comparison of pressure coefficients with the published test results. The LES and FW-H are applied to obtain the characteristic acoustic pressure spectra, and the acoustic sources are identified using the Lamb vector of the vortex theory. Inherently, the flexible spokes are the main aerodynamic noise source
2. In order to control flow and reduce aerodynamic noise, a bionic nonsmooth riblet surface structure is arranged on the spokes of the NPT. The influence of the position, direction, and size of the riblet structures on aerodynamic noise was analyzed, and it is evident that, by appropriating the exact parameters (position and size), a 5.19 dB noise reduction can be achieved
3. From the perspective of the vortex theory, the mechanism of reducing noise is the nonsmooth riblet structures significantly decreasing the Lamb vector near the spokes and breaking the band-like distribution of the Lamb vector at the spoke edges and thus the aerodynamic noise of NPT. The result is worthwhile for the design of a low aerodynamic noise NPT
4. However, the effects of deformation of the NPT spokes, wheel eyebrow, vehicle structure, and other structures on the aerodynamic noise of the tire are not considered. In addition, the riblet size parameters given herein may not be optimal. These issues provide avenues for further future research

## Figures and Tables

**Table 1:** Dimensional parameters of various parts of NPT.

Part name	Size/quantity
Shear outer diameter	593 mm
Hub diameter	410 mm
Tire width	195 mm
Shear band thickness	19.5 mm
Number of spokes	50 (25 couples)
Spoke thickness	4.2 mm
Spoke length	72 mm

**Table 2:** Grid quality parameters used for sensitivity analysis.

Grid schemes	Maximum mesh size on tire surface (mm)	First layer boundary layer thickness (mm)	Number of boundary layers	Total number of grids	100 steps time (sec)
Grid 1	6	0.2	3	2,189,794	270.3
Grid 2	4	0.2	3	2,894,271	342.1
Grid 3	3	0.15	4	5,789,238	689.9
Grid 4	2	0.1	5	11,749,048	1,416.5

**Table 3:** Position coordinates of sound measuring points.

Measuring point number	*X* coordinate (m)	*Y* coordinate (m)	*Z* coordinate (m)
1	1.0	0.1	0
2	1.2929	0.1	0.7071
3	2.0	0.1	1.0
4	2.7071	0.1	0.7071
5	3.0	0.1	0

**Table 4:** SPL at each measuring point in the original model.

Measuring point number	SPL (dB)
1	63.0843
2	62.2977
3	60.3082
4	59.5186
5	68.6636
Total SPL	71.1566

**Table 5:** Dimensional parameters of various arrangements of riblet structures.

Scheme number	Arrangement area	Arrange direction	*N*_*L*_	*N*_*R*_	*N*_*θ*_
A	Spoke	Landscape		12	
B	Spoke	Radial	16		
C	Shear band, hub	Landscape			5
D	Shear band, hub	Circumference	16		
E	(A and C combination)			12	5
F	(A and D combination)		16	12	
G	(B and C combination)		16		5
H	(B and D combination)		16		

**Table 6:** Changes in SPL with various riblet structure schemes.

Scheme number	Total SPL (dB)	Δ*L*_*p*_
Original model	71.1566	—
A	68.3390	-2.8176
B	65.9669	-5.1897
C	76.1547	4.9981
D	70.2097	-0.9469
E	68.8993	-2.2573
F	67.6845	-3.4721
G	72.8036	1.6470
H	66.9558	-4.2008

**Table 7:** Changes in SPLs with carious riblet dimensions.

Scheme number	Triangular cross section length (mm)	Riblet pitch (mm)	*N*_*L*_	Total SPL (dB)	Δ*L*_*p*_ (dB)
Original model	—	—	—	71.1566	—
B1	1	2.00	48.5	74.7221	3.5655
B2	2	4.02	24	70.5889	-0.5677
B3	3	6.00	16	65.9669	-5.1897
B4	4	7.96	12	66.1336	-5.0230
B5	5	10.0	9.5	71.6285	0.4719

## Data Availability

The data used to support the findings of this study are available from the corresponding author upon request.
